# Cyanidin-3-*O*-Glucoside Protects against 1,3-Dichloro-2-Propanol-Induced Reduction of Progesterone by Up-regulation of Steroidogenic Enzymes and cAMP Level in Leydig Cells

**DOI:** 10.3389/fphar.2016.00399

**Published:** 2016-11-04

**Authors:** Jianxia Sun, Wei Xu, Cuijuan Zhu, Yunfeng Hu, Xinwei Jiang, Shiyi Ou, Zhijian Su, Yadong Huang, Rui Jiao, Weibin Bai

**Affiliations:** ^1^Faculty of Chemical Engineering and Light Industry, Guangdong University of TechnologyGuangzhou, China; ^2^Department of Food Science and Engineering, Institute of Food Safety and Nutrition, Jinan UniversityGuangzhou, China

**Keywords:** cyanidin-3-*O*-glucoside, 1,3-DCP, progesterone, cAMP, StAR, mitochondrial membrane potential

## Abstract

1,3-Dichloro-2-propanol (1,3-DCP) is a food processing contaminant and has been shown to perturb male reproductive function. Cyanidin-3-*O*-glucoside (C3G), an anthocyanin antioxidant, is reported to have protective effects on many organs. However, it remains unclear whether C3G protects against chemical-induced reproductive toxicity. The present study was therefore to investigate the intervention of C3G on 1,3-DCP-induced reproductive toxicity in R2C Leydig cells. Results demonstrated that C3G inhibited the 1,3-DCP-induced cytotoxicity and cell shape damage with the effective doses being ranging from 10 to 40 μmol/L. In addition, 1,3-DCP (2 mmol/L) exposure significantly increased the ROS level and mitochondrial membrane potential damage ratio, leading to a decrease in progesterone production, while C3G intervention reduced the ROS level, and increased the progesterone production after 24 h treatment. Most importantly, C3G intervention could up-regulate the cyclic adenosine monophosphate (cAMP) level and protein expression of steroidogenic acute regulatory protein and 3β-hydroxysteroid dehydrogenase. It was concluded that C3G is effective in reducing 1,3-DCP-induced reproductive toxicity via activating steroidogenic enzymes and cAMP level.

## Introduction

In recent years, food chemical contaminants have attracted more and more attention. 1,3-Dichloro-2-propanol (1,3-DCP) is one of them. It is produced in the processing, cooking, and storage of many foodstuffs such as soy sauce, oyster sauce, acid-hydrolyzed vegetable proteins, and processed meat products due to reaction of chloride ions with lipid components in foods (Velisek et al., 1978; Kim et al., 2015). Besides, 1,3-DCP is used in the manufacture of epichlorohydrin, a flocculant for water purification, so 1,3-DCP also exists in water as a contaminant (Cal/EPA, 2010). The Joint FAO/WHO Expert Committee on Food Additives (JECFA) has concluded that 1,3-DCP is a genotoxic carcinogen, and leads to tumor development in various organs. So far, epidemiological data are still unavailable, anda tolerable daily intake for 1,3-DCP has not been established (The Joint FAO-WHO Expert Committee Report on Food Additives [JECFA], 2001). According to the JECFA assessment, mean and 95th percentile dietary exposure estimates for 1,3-DCP in various foods were 0.008–0.051 μg/kg bw/day and 0.025–0.136 μg/kg bw/day, respectively, in population (The Joint FAO-WHO Expert Committee Report on Food Additives [JEFCA], 2007). Due to its good water-solubility, 1,3-DCP is widely distributed in the body fluids and can cross the blood–brain barrier and blood-testis barrier easily (Edwards et al., 1975; Yamada et al., 2010). Reproductive toxicity of 1,3-DCP has been investigated in several animal studies (Omura et al., 1995; Lee et al., 2009; Andres et al., 2013), suggesting it may cause the sperm deformity in epididymis and a reduction of sperm count in male rats (Omura et al., 1995). In our previous study, 1,3-DCP was found to reduce the progesterone production by inhibiting the activity of steroidogenic enzymes and decreasing cAMP level in Rat Leydig cells (R2C) (Sun et al., 2014).

Anthocyanins, a largest group of water-soluble pigments are rich in many fruits, such as blueberry, strawberry, wolfberry, mulberry, blackberry, and grape (Sun et al., 2010, 2011a). Numerous studies have demonstrated that anthocyanins possess activities of antioxidant, anti-atherosclerosis, anti-tumor, and anti-inflammatory (Mazza, 2007; Jiao et al., 2010; Sun et al., 2011b; Skrovankova et al., 2015; Hu et al., 2016; Lila et al., 2016). It has been reported that the daily consumption of anthocyanins is 180–255 mg in the USA diet, higher than the intake of most other flavonoids (McGhie and Walton, 2007). Among a variety of anthocyanins, cyanidin-3-*O*-glucoside (C3G) is the most common and abundant one in fruits (Castaneda-Ovando et al., 2009). It has been reported that C3G are neuro-, hepato-, and cardio-protective (Speciale et al., 2010; Min et al., 2011; Zhu et al., 2012; Qin et al., 2013). However, it remains unclear whether anthocyanins protect against chemical-induced reproductive toxicity. Due to its ability of synthesizing and secreting a great amount of progesterone without hormonal stimulation, rat Leydig cells (R2C) is often employed as an *in vitro* model for male reproductive toxicity study (Rao et al., 2003). The present study was therefore to examine the protective effect of C3G against the 1,3-DCP-induced male reproductive toxicity and the associated underlying mechanism in R2C cells.

## Materials and Methods

### C3G Extraction

Cyanidin-3-*O*-glucoside was extracted from mulberry. The mulberry was purchased from Guangdong Academy of Agricultural Sciences, and dried in an oven at a low temperature about 60°C. Dried mulberry was soaked in 24 volumes of 60% ethanol containing 0.1% trifluoroacetic acid for 2 h and then homogenized. Ultrasonic Cell Disruptor was used to assist the extraction. The extraction was repeated for three times. All the extracts were mixed and the ethanol was removed at 42°C in a rotary evaporator. The crude extract was then partitioned against petroleum ether to remove the lipid components. Then the aqueous extract was purified on an Amberlite XAD-7HP resin column (Rohm and Haas, Philadelphia, PA, USA), and freeze-dried. The resultant powder was rich anthocyanins. C3G was purified using a middle-pressure liquid chromatography (MPLC, Labomatic, Switzerland) with C^18^ adsorption column, the fraction containing C3Gwas then lyophilized and stored under -20°C until use.

### HPLC-DAD Analysis of C3G

**FIGURE 1:**
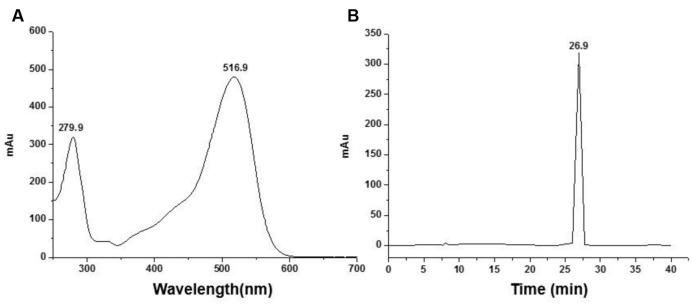
**Identify the purity of Cyanidin-3-*O*-glucoside (C3G) by HPLC-DAD. (A)** Full wave scanning of C3G. **(B)** 280 nm wave scanning for purity identification.

HPLC-DAD analysis was performed in a Water-DAD system. C3G sample was injected into a Venusil ASB-C^18^ HPLC column (250 × 4.6 mm; i.d., 5 μm; Agela Technologies) with a DAD detector at 280 and 560 nm with the column temperature being maintained at 30°C. The gradient mobile phase composed of solvent A (5% formic acid) and solvent B (formic acid: water: methanol, 5:45:50; v/v/v). Gradient elution was programmed at first, the ratio of A to B was 60:40, 0–3 min, at a flow rate of 0.4 mL/min; then changed to linear gradient elution, B increased from 40 to 60% in 24 min, at a flow rate of 0.4 mL/min; B increased from 60 to 100% in 5 min, at a flow rate of 0.8 mL/min; finally B decreased to 40% and held for additional 8 min, and the whole program lasted for 40 min. From the results of the full wave scanning (**Figure 1A**), C3G has two absorption peaks at 280 and 517 nm, respectively, just as the characters of anthocyanins published before. Compared to the peak area and the sum of relative peak area (**Figure 1B**), the purity of C3G was higher than 95%.

### Cell Culture

The Rat Leydig cells (R2C), obtained from the ATCC (Manassas, VA, USA), were cultured in DMEM-F12 medium (Gibco, Rockville, MD, USA) supplemented with sodium pyruvate, NaHCO_3_, 15% horse serum (HS), 2.5% fetal bovine serum (FBS), and a 1% penicillin/streptomycin mixture. The cells were incubated at 37°C with 5% CO_2_.

### Cell Viability Assay

MTT assay was used to evaluate cell viability. The R2C cells were seeded in 96-well plates (Costar, Cambridge, MA, USA) at a density of 4 × 10^4^ cells per well, cultured at 37°C for 24 h. First, the cells were treated with the designated concentrations of 1,3-DCP (0, 1, 2, 4, 6, and 8 mmol/L) for 24 h, then added with 10 μL of MTT (5 mg/mL in PBS). After continued incubation at 37°C for additional 4 h, the medium was removed, 200 μL DMSO was added to each well and mixed for 10 min at room temperature. The absorbance was measured using a microplate reader (Thermo Scientific, Chantilly, VA, USA) at 570 nm. In addition, cell viability assay was also performed in R2C cells disposed with various concentrations of C3G (0, 1, 5, 10, 20, 50, and 100 μmol/L), or 2 mmol/L 1,3-DCP with or without various concentrations of C3G. Data were then analyzed through Graphpad software.

### Cell Morphological Observation

For morphological observation, the R2C cells were cultured in six-well plates at a density of 2 × 10^5^ for 24 h, then treated with 2 mmol/L 1,3-DCP with or without C3G (5, 10, 20, and 40 μmol/L) for 24 h. The protective effect of C3G on 1,3-DCP-induced cell shape damage was observed using a fluorescence microscope (Olympus. ×71).

### Apoptosis Analysis

R2C cells were cultured in six-well plates at a density of 2 × 10^5^ for 24 h. Treated cells were harvested and washed with PBS, and centrifuged at 300 *g* for 5 min at room temperature. The supernatant was discarded and the cells were resuspended in 200 μl Binding Buffer. Apoptosis was measured using Annexin V-FITC kit (Abcam) following the manufacturer’s instructions and analyzed by flow cytometry (BD FACSaria, USA).

### Radioimmunoassay for Measurement of Progesterone Levels

R2C cells were disposed with 2 mmol/L 1,3-DCP with or without various concentrations of C3G (5, 10, 20, and 40 μmol/L) for 4 and 24 h. Progesterone levels in the culture media were measured by a radioimmunoassay kit (Beijing North Institute of Biological Technology, Beijing, China) according to the manufacturer’s instructions.

### Determination of Reactive Oxygen Species (ROS) Production

After the treatment, cells were washed with PBS, and fixed with 1 ml Dichloro-dihydro-fluorescein diacetate (DCFH-DA) solution (10 μmol/L) at 37°C for 30 min. Then the cells were washed three times with PBS and trypsinized to a single cell suspension. Cellular fluorescence was measured using a fluorometric plate reader at an excitation/emission of 488 nm/525 nm.

### Measurement of Mitochondrial Membrane Potential (MMP)

The JC-1 (Beyotime Biotech, Nantong, China) staining method was used to measure the mitochondrial membrane potential (MMP; ΔΨm) of R2C cells following the manufacturer’s instructions. In brief, the treated cells were cultured in six-well plates and disposed with JC-1 staining solution (5 μg/mL) for 20 min at 37°C, and rinsed twice with JC-1 staining buffer and analyzed on a flow cytometer (BD FACSaria, USA) using 488 nm excitation with 670 nm emission filters.

### Western Blot Analysis

Total proteins were extracted according to the method described previously by Sun et al. (2014). In brief, cells were lysed in RIPA lysis buffer (Cell Signaling, Beverly, MA, USA) on ice, the supernatants were collected after centrifugation at 12,000 *g* for 15 min at 4°C. Protein concentration was measured by BCA assay. Total proteins were separated on a 12% SDS-polyacrylamide gel and transferred to polyvinylidene difluoride membranes. Membranes were then blocked for 1–2 h at room temperature and then incubated at 4°C overnight using relative primary antibodies: rabbit anti-rat StAR antibody (Santa Cruz Biotechnology, Santa Cruz, CA, USA) at 1:1000, rabbit anti-rat 3β-HSD antibody (Santa Cruz Biotechnology) at 1:500, and rabbit anti-rat GAPDH antibody (Cell Signal Technology, Beverly, MA, USA) at 1:1000. The membranes were then incubated for 2 h at room temperature in diluted horseradish peroxidase-conjugated secondary antibody, and detected with SuperSignal West Pico Chemiluminescent Substrate (Thermo Scientific, Rockford, IL, USA) following the manufacturer’s protocol.

### Measurement of cAMP Accumulation

Cyclic adenosine monophosphate (cAMP) accumulation in cells was determined using cAMP-Glo assay kit (Promega, Madison, WI, USA). In brief, R2C cells were plated in F12 medium on 96-well dishes at a density of 1 × 10^4^ for 24 h. The cells were disposed with 2 mmol/L 1,3-DCP with or without various concentrations of C3G (5, 10, 20, and 40 μmol/L) for 4 h. After 20 min of treatment with induction buffer [PBS containing the phosphodiesterase inhibitors IBMX (0.5 mM) and Ro 20-1724 (0.1 mM); Sigma–Aldrich, St. Louis, MO, USA], the reaction was stopped by cAMP-Glo Lysis Buffer. Then, the kinase reaction was performed using 40 μL cAMP Detection Solution for 20 min. At the end of the kinase reaction, 80 μL Kinase-Glo Reagent was added and incubated for 10 min, then a plate-reading luminometer (Biotek, Synergy HT, Winooski, VT, USA) was used for measurement.

### Statistical Analysis

Values are means of triplicate measurements for all experiments. The significance of difference was assessed using one-way ANOVA. *p* < 0.05 was considered significant in statistic.

## Results

### The Cell Viability of R2C under Different Interventions

**FIGURE 2:**
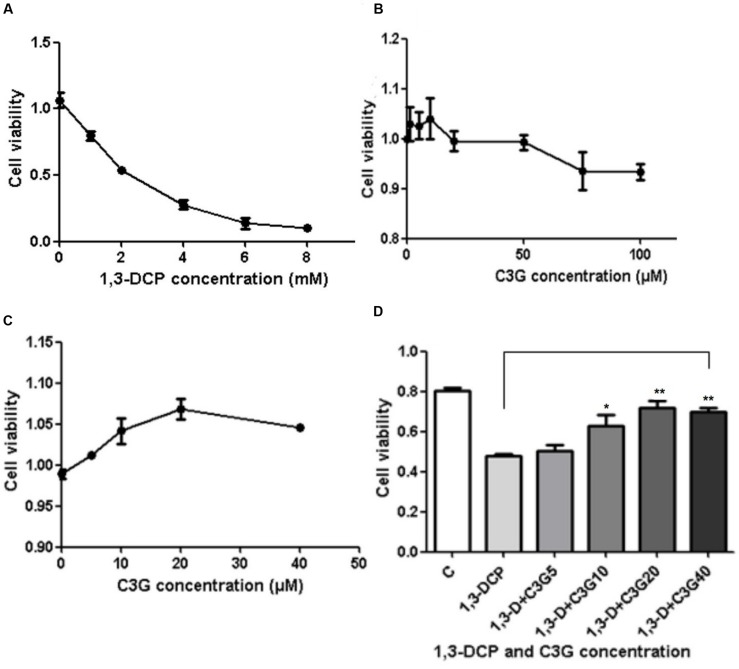
**Effects of C3G and 1,3-Dichloro-2-propanol (1,3-DCP) intervention on cell viability ratio in R2C cells. (A)** Cell viability ratio of R2C cells disposed with various concentrations of 1,3-DCP (0, 1, 2, 4, 6, and 8 mmol/L) for 24 h. **(B)** Cell viability ratio of R2C cells disposed with C3G at the concentrations of 0, 1, 5, 10, 20, 50, and 100 μmol/L or **(C)** 0, 10, 20, and 40 μmol/L. **(D)** Cell viability ratio of R2C cells disposed with 2 mmol/L with or without various concentrations of C3G (0, 10, 20, and 40 μmol/L). Values were represented as Mean ± SD of three independent experiments. ^∗^*p* < 0.05 versus 1,3-DCP group, ^∗∗^*p* < 0.01 versus 1,3-DCP group.

The cell viability decreased with the increase of 1,3-DCP concentration of 0, 1, 2, 4, 6, and 8 mmol/L (**Figure 2A**) At the dosage of 2 mmol/L, the cell viability decreased to 48.7%. Therefore, 2 mmol/L was chosen as the proper concentration of 1,3-DCP for R2C cell injury model in further experiments. In addition, cytotoxicity of C3G was also determined, C3G showed protective effect on R2C cell viability at the concentrations of 0–40 μM (**Figures 2B,C**). Then the R2C cells were disposed with 2 mmol/L 1,3-DCP with or without various concentrations of C3G (5, 10, 20, and 40 μM) for 24 h. The results showed that C3G intervention, especially at the concentration of 20 and 40 μM, significantly reversed 1,3-DCP -induced cytotoxicity in R2C cells (**Figure 2D**).

### Changes in Cell Morphological and Cell Apoptosis

**FIGURE 3:**
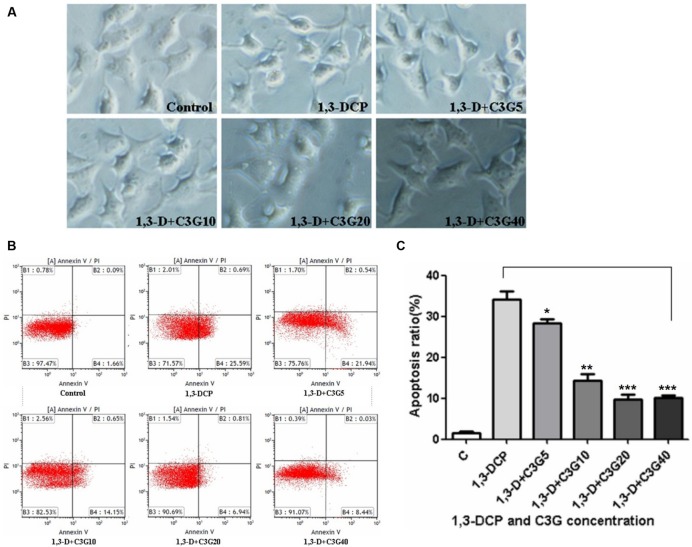
**Effects of C3G and 1,3-DCP intervention on cell shape and apoptosis in R2C cells. (A)** Cell shape disposed with various C3G and 1,3-DCP concentrations (400 ×). **(B)** Cell apoptosis treated with various C3G and 1,3-DCP concentration. **(C)** Cell apoptosis ratio exposed to various C3G and 1,3-DCP concentrations. Values were represented as Mean ± SD of three independent experiments. ^∗^*p* < 0.05 versus 1,3-DCP group, ^∗∗^*p* < 0.01 versus 1,3-DCP group, ^∗∗∗^*p* < 0.001 versus 1,3-DCP group.

The morphological changes of the cell surface were observed (**Figure 3A**). When treated with 2 mmol/L 1,3-DCP, the R2C cells were becoming shrinking and floating, and the filamentous-shaped cells increased. After intervention with various concentration of C3G, especially 20 and 40 μmol/L, the cell morphology changed less compared with the control. The cell morphological changes were consistent with cell viability data.

Cell apoptosis was detected by flow cytometry. 1,3-DCP significantly increased the percentage of apoptotic cells. After intervention with various concentrations of C3G, especially 10–40 μmol/L, the apoptosis ratio decreased significantly (**Figures 3B,C**). Thereby it was further proved that C3G could protect against 1,3-DCP-induced R2C cell injury.

### Changes in Progesterone Secretion

**FIGURE 4:**
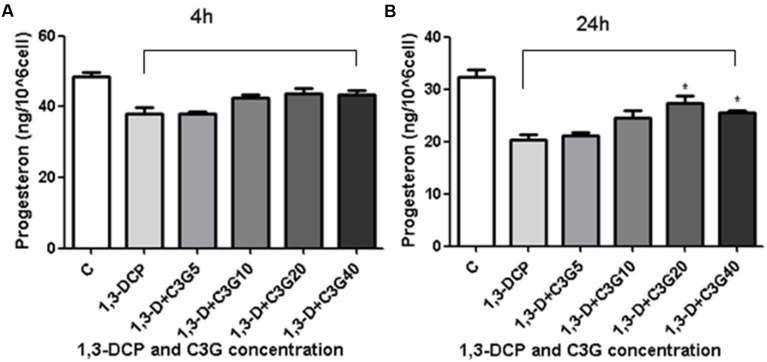
**Effects of C3G and 1,3-DCP intervention on progesterone production in R2C cells. (A)** Progesteron production of R2C cells disposed with 2 mmol/L 1,3-DCP with or without various concentrations of C3G (5, 10, 20, and 40 μmol/L) in 4 h or **(B)** 24 h. Values were represented as Mean ± SD of three independent experiments. ^∗^*p* < 0.05 versus 1,3-DCP group.

After treatment with 2 mmol/L 1,3-DCP for 4 h, the progesterone production of R2C cells decreased compared with control, but C3G intervention could not significantly increase the progesterone secretion at the same time. However, after 24 h treatment, the addition of C3G (20–40 μmol/L) significantly increased the progesterone secretion in 1,3-DCP treated cells. The data suggested that C3G protected against 1,3-DCP-induced reduction of progesterone synthesis in a time- and dose-dependent manner (**Figure 4**).

### Changes in ROS Production

**FIGURE 5:**
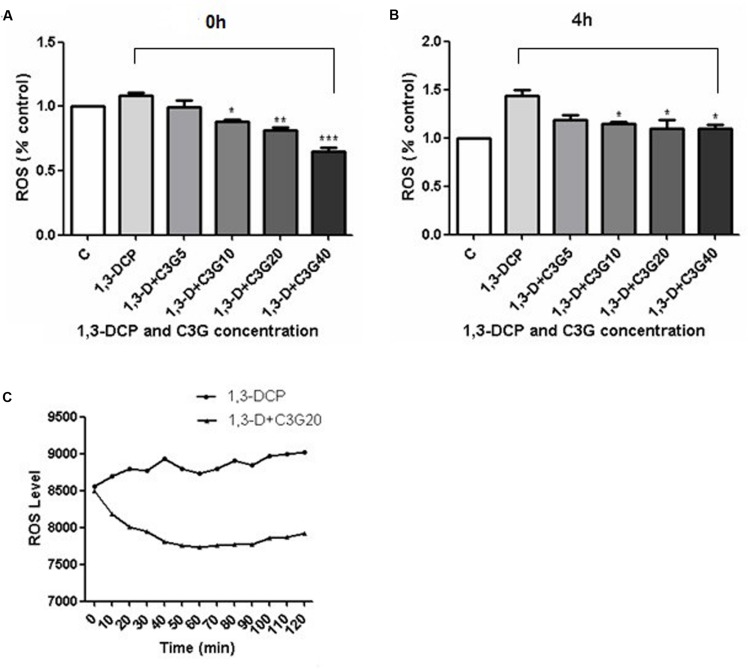
**Effects of various concentrations of C3G and 1, 3-DCP on ROS level at different time. (A)** ROS generation (% control) of R2C cells disposed with 2 mmol/L 1,3-DCP with or without various concentration of C3G (5, 10, 20, and 40 μmol/L) immediately or **(B)** in 4 h. **(C)** ROS level of R2C cells disposed with 2 mmol/L 1,3-DCP with or without 20 μmol/L C3G from 0–120 min. Values were represented as Mean ± SD, *n* = 3. ^∗^*p* < 0.05 versus 1,3-DCP group, ^∗∗^*p* < 0.01 versus 1,3-DCP group, ^∗∗∗^*p* < 0.001 versus 1,3-DCP group.

The results showed that ROS generation increased immediately after 1,3-DCP treatment, and much more increased after 4 h treatment (**Figures 5A,B**). The addition of C3G, ranging from 10 to 40 μmol/L, reduced the ROS production significantly in 1,3-DCP treated cells immediately or 4 h later. Furthermore, the ROS level was also examined in 1,3-DCP-treated cells with or without 20 μmol/L C3G from 0 to 120 min (**Figure 5C**). The data showed that 20 μmol/L C3G obviously reduced the ROS level at different time points, while a rising trend of ROS was observed in C3G supplement group from 90 to 120 min.

### Changes in the MMP and cAMP Levels

**FIGURE 6:**
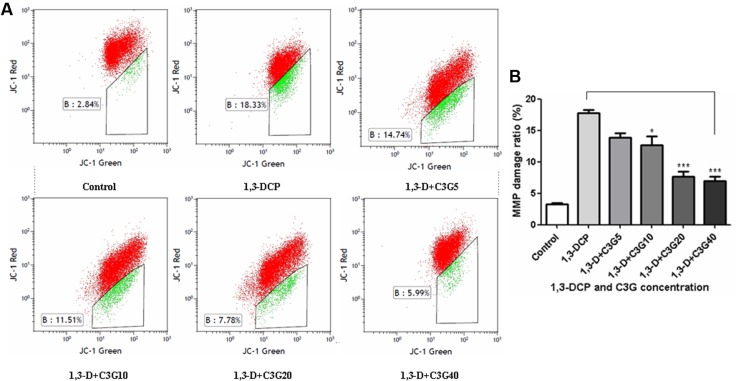
**Effects of C3G and 1,3-DCP intervention on mitochondrial membrane potential (MMP) changes in R2C cells. (A)** JC-1 dye was used as an indicator of MMP in R2C cells. The MMP level was detected by flow cytometry, JC-1 aggregate image was represented by red fluorescence, and JC-1 monomer was represented by green fluorescence, **(B)** MMP damage ratio (or called mitochondrial depolarization) was indicated by green/red fluorescence intensity ratio. Values were represented as Mean ± SD of three independent experiments. ^∗^*p* < 0.05 versus 1,3-DCP group, ^∗∗∗^*p* < 0.001 versus 1,3-DCP group.

JC-1 dye was used as an indicator of MMP in R2C cells. The MMP level was detected by flow cytometry, JC-1 aggregate image was represented by red fluorescence, and JC-1 monomer was represented by green fluorescence, mitochondrial depolarization was indicated by green/red fluorescence intensity ratio (or called MMP damage ratio). The results showed that 1,3-DCP increased the MMP damage ratio from 2.84 to 18.33%, and addition of C3G decreased the ratio in a dose-dependent manner (**Figure 6**).

**FIGURE 7:**
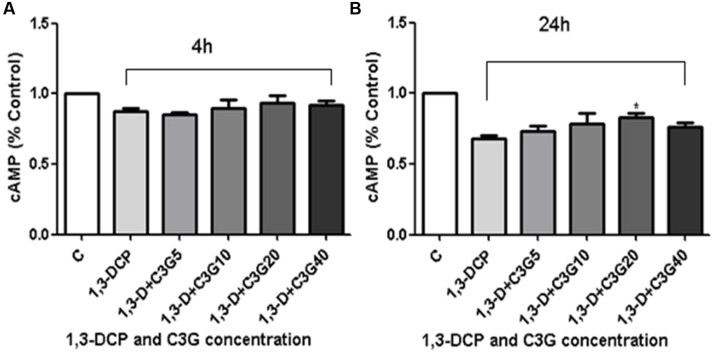
**Effects of C3G and 1,3-DCP intervention on cAMP levels in R2C cells. (A)** cAMP level (% control) of R2C cells disposed with 2 mmol/L 1,3-DCP with or without various concentration of C3G (5,10, 20, and 40 μmol/L) in 4 h or **(B)** 24 h. Values were represented as Mean ± SD of three independent experiments. ^∗^*p* < 0.05 versus 1,3-DCP group.

Cyclic adenosine monophosphate plays an important role in progesterone synthesis, there is also a positive correlation between cellular cAMP level and androgen production. The results showed that the 1,3-DCP treatment alleviated the intracellular cAMP level after 24 h treatment, but not obviously after 4 h. Addition of various concentrations of C3G, especially 20 μmol/L, increased cAMP level after 24 h (**Figures 7A,B**).

### Immunoblot Analyses of StAR and 3β-HSD

**FIGURE 8:**
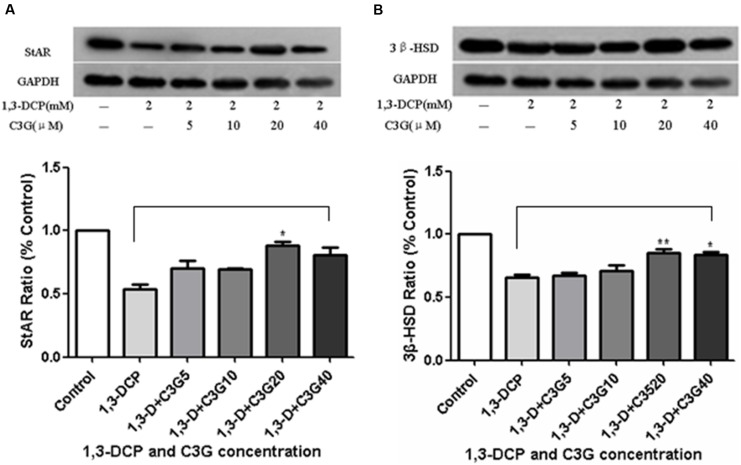
**Effects of C3G and 1,3-DCP intervention on protein expression levels of StAR and 3β-HSD in R2C cells. (A)** Protein expression level of StAR (% control) of R2C cells disposed with 2 mmol/L 1,3-DCP with or without various concentration of C3G (5, 10, 20, and 40 μmol/L), **(B)** Protein expression level of 3β-HSD (% control) of R2C cells disposed with 2 mmol/L 1,3-DCP with or without various concentration of C3G (5, 10, 20, and 40 μmol/L). Values were represented as Mean ± SD of three independent experiments. ^∗^*p* < 0.05 versus 1,3-DCP group, ^∗∗^*p* < 0.01 versus 1,3-DCP group.

StAR and 3β-HSD are the key proteins in progesterone synthesis. As depicted in **Figure 8**, 1,3-DCP down-regulated the protein levels of StAR and 3β-HSD, the addition of C3G reversed the trend, especially 20 μmol/L C3G up-regulated both StAR and 3β-HSD protein levels significantly.

## Discussion

1,3-Dichloro-2-propanol-induced reproductive toxicity has become an issue of health concerns. In our previous study, 1,3-DCP decreased the cAMP level and MMP in a dose-depend manner in R2C cells because it caused an increase of ROS generation, resulting in the mitochondrial dysfunction, progesterone biosynthesis inhibition and R2C cell apoptosis (Sun et al., 2014). The present results clearly demonstrated that C3G could protect against 1,3-DCP-induced reproductive toxicity in R2C cells.

Leydig cells is able to synthesize and secrete the testosterone in the male testes. It is well known that progesterone is the precursor of testosterone and therefore regarded as the key evaluation index of reproductive function in R2C cells. Like most cells, R2C cells generate ROS during normal energy metabolism, and mitochondria are the major source of intracellular ROS. Several researches have demonstrated that mitochondrial steroidogenesis is sensitive to oxidative stress in Leydig cells (Hales, 2002; Diemer et al., 2003; Allen et al., 2004). Oxidative stress could perturb mitochondria, leading to an abrupt cessation to cholesterol transfer and steroid hormone production Leydig cells (Allen et al., 2004). Therefore, when R2C cells are exposure to chemicals, like 1,3-DCP and 3-Monochloropropane-1,2-diol (3-MCPD), ROS production becomes excessive, resulting in mitochondrial dysfunction, cell apoptosis, and lesser progesterone production (Sun et al., 2013, 2014). In the present study, C3G intervention could reduce the ROS generation immediately and continually within 4 h. Results on the MTT assay and observation on cell apoptosis and morphological changes demonstrated that C3G could reverse the 1,3-DCP-induced cell injury and cell apoptosis in a dose-depend manner. It was apparent that C3G could suppress ROS generation and restore the mitochondria function. Results showed that the longer the 1,3-DCP exposure was, the higher ROS level was. When C3G was added into the culture, the ROS decreased and consequently the progesterone level increased obviously after 24 h treatment. Therefore, the protective effect of C3G on progesterone production requires a certain amount of time to attenuate 1,3-DCP induced ROS generation and cell injury in R2C cells.

Cyanidin-3-*O*-glucoside could up-regulate the protein levels of steroidogenic enzymes, StAR and 3β-HSD, as well as cAMP. It is well known that mitochondria are the key point for steroid hormone biosynthesis. The transfer of cholesterol from the cytoplasm into mitochondria inner membrane is the first rate-limiting step, which is initiated via StAR protein. Then cholesterol is converted to pregnenolone in the mitochondria matrix by a cytochrome P450 side-chain cleavage enzyme (P450scc, CYP11A). Pregnenolone diffuses to the smooth endoplasmic reticulum and is further converted into progesterone via 3β-HSD, 17α-hydroxylase (P450c17, CYP17), and 17β-hydroxysteroid dehydrogenase type III (17HSD3) (Payne and Hales, 2004). The present study clearly demonstrated that 1,3-DCP disrupted mitochondria function, suppressed StAR protein level, while C3G at the concentration of 20 μmol/L significantly up-regulated StAR and 3β-HSD expression in the Leydig cells and partially restored normal progesterone reproduction (Rasmussen et al., 2013). Addition of C3G (20–40 μmol/L) could significantly increase 3β-HSD protein level. Therefore, this confirmed that C3G could protect against 1,3-DCP-induced mitochondria dysfunction. On the other hand, testosterone production by Leydig cells is mainly regulated by luteinizing hormone (LH), which acts via cAMP to regulate testosterone production. cAMP, as the second messenger, can activate protein kinase A (PKA) and initiate steroid hormone biosynthesis by StAR protein (Hales et al., 2005). Zhu et al. (2012) found that C3G could attenuate hyperglycemia-induced hepatic oxidative damage by activating glutathione synthesis through cAMP-PKA-dependent signaling pathway. In the same way, the present results demonstrated that 1,3-DCP suppressed the cAMP level after 24 h treatment in R2C cells, while C3G intervention restored the cAMP level, leading o up-regulation of StAR expression and and steroid hormone synthesis.

## Conclusion

**FIGURE 9:**
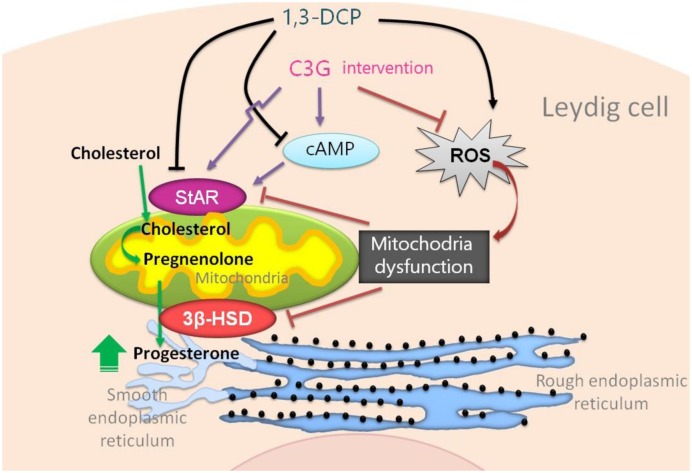
**Cyanidin-3-*O*-glucoside protection mechanism against 1,3-DCP-induced reduction of progesterone in R2C cells**.

Cyanidin-3-*O*-glucoside intervention increased progesterone production in 1,3-DCP-treated R2C cells by modulating the cAMP, MMP, and expression level of steroidogenic enzymes. C3G also protected against 1,3-DCP-induced cell morphological changes and apoptosis in R2C cells (**Figure 9**).

## Author Contributions

JS designed the experiments and performed the experiment. WX and CZ performed the experiments. YuH and XJ contributed reagents/materials/analysis tools. SO performed the data analysis. ZS and YaH helped perform the analysis with constructive discussions. RJ wrote the paper. WB conceived and designed the experiments.

## Conflict of Interest Statement

The authors declare that the research was conducted in the absence of any commercial or financial relationships that could be construed as a potential conflict of interest.
